# Research Progress on Titanium–Niobium Micro-Alloyed High-Strength Steel

**DOI:** 10.3390/ma18020325

**Published:** 2025-01-13

**Authors:** Yunshuang Zhang, Yuxi Ma

**Affiliations:** School of Civil and Architectural Engineering, Wuhan Polytechnic University, Wuhan 430023, China; 20220812019@whpu.edu.cn

**Keywords:** Ti-Nb micro-alloying, rolling technology, fine-grain strengthening, precipitation strengthening

## Abstract

Research on micro-alloyed steel is a strategic measure to meet the needs of various industries and promote green development, and it is essential for many major steel-producing countries. Currently, the mainstream micro-alloying elements in the research and application of micro-alloyed steel are V, Ti, and Nb. Due to the high price of V, the actual production is mostly achieved by adding titanium–niobium composite to change the properties of high-strength steel. This article begins by examining the strengthening mechanisms in titanium–niobium micro-alloyed high-strength steel. It then reviews the literature on how metallurgical processes and second-phase particles affect the steel’s properties. The article summarizes the current research status and analyzes the problems in the existing research process and results. Finally, it explores future research directions, offering insights into subsequent studies and applications.

## 1. Introduction

Steel materials are the fundamental materials on which global society and economy rely for development and are also the most commonly used in industrial production and people’s lives. Today, steel still dominates structural materials because of its highly competitive advantages. In the development of the world, this position will remain unshakable. The applications of micro-alloying technology, which include precipitation strengthening and grain refinement, have effectively improved the strength and the processing performance of steel materials. It is an emerging metallurgical technology that appeared in the 1970s and is a major step in the development of human industrial history. In the following decades, it has become a research hotspot and greatly developed [1,2,3,4]. At present, the most important micro-alloying elements for steel research and application are Nb, V, and Ti; the alloying of V mainly enhances the precipitation strengthening effect, while the fine-grain strengthening effect is weak. It is difficult to achieve the desired effect by adding V alone. Generally, V is added in combination with Nb and Ti. Due to the high price and economic cost of V, using a composite addition of Nb Ti micro-alloying elements instead of V addition can reduce production costs at the same strength and ductility levels. The performance stability of products with Ti-Nb micro-alloying element composite addition is greatly improved compared to products with Ti added alone while also saving a large amount of Nb element.

The use of micro-alloying technology to add trace amounts of Ti, Nb, and other elements to steel can not only improve its strength and toughness but also reduce manufacturing costs. There are two main strengthening methods for micro-alloying elements in steel: solid solution strengthening and second-phase particle strengthening. Other studies have shown that micro-alloying elements can also refine grain size, thereby improving the strength of steel. In recent years, as equipment manufacturing has developed towards large-scale, high-efficiency, and lightweight applications, the performance requirements for steel materials have increased. Developing high-performance steel materials has enormous economic and social benefits. Improving comprehensive performance and extending the service life of steel materials are key tasks for enhancing the competitiveness of the national manufacturing industry [5,6]. Micro-alloy technology, as a technical means to improve the comprehensive performance of steel materials, is particularly important.

In the current field of materials science, research on high-strength steel is of significant importance, particularly in seeking a balance between enhancing material performance and reducing production costs. Although a substantial body of literature addresses the application of micro-alloying techniques in steel, there remain key issues and challenges in the research area of titanium-niobium micro-alloyed high-strength steel that have not been adequately resolved in the existing literature. This review article aims to provide a comprehensive perspective, offering novel insights in several areas: Firstly, we conduct an integrated analysis of the latest research findings in the field of titanium-niobium micro-alloyed high-strength steel, including microstructure control, mechanical property optimization, and environmental adaptability evaluation; secondly, we pay special attention to the latest metallurgical technologies and processing techniques, such as controlled rolling and rapid cooling, which show great potential in enhancing material performance and reducing production costs; thirdly, this review emphasizes the dual benefits of titanium-niobium micro-alloyed steel in achieving green development and reducing production costs, aligning with the current global trend of sustainable development; finally, based on an in-depth analysis of existing research, we propose new directions for future research, including intelligent manufacturing and data-driven material design, which are frontier topics driving the development of this field. Therefore, this review not only provides the research community with a systematic update of knowledge but also offers new research ideas and collaboration opportunities for researchers in materials science, metallurgical engineering, and related fields, expecting to positively impact the research and application of titanium-niobium micro-alloyed high-strength steel and provide guidance for future research directions.

## 2. Application and Problems of Titanium–Niobium Micro-Alloyed Steel

### 2.1. Development Process of Micro-Alloyed Steel

Compared to ordinary carbon steel and low-alloy steel, micro-alloyed steel has many unique characteristics. The introduction of the concept of micro-alloying elements can be traced back to the 1920s when Field and Beckett first reported the effect of trace zirconium addition on the properties of traditional carbon structural steels [7]. At the same time, titanium began to be used as an element to improve the welding performance of steel and suppress the excessive growth of austenite grains in the welding heat-affected zone [8]. In the 1950s, Hall–Petch established the correlation between material mechanical properties and grain size, indicating that grain refinement not only enhances strength but also increases material toughness. In the 1960s, research by scholars such as Davenport [9] further revealed that micro-alloyed steel can form nanoscale precipitation particles under specific process conditions, which have significant precipitation strengthening effects. The fine-grain strengthening mechanism and precipitation strengthening mechanism constitute the core strengthening theory of micro-alloyed steel.

In the 1970s, the “Micro-alloying 75” conference held in the United States [10,11,12,13,14] summarized the theory and application achievements of micro-alloyed steel and clarified the research direction of micro-alloyed steel. In the 1980s, breakthrough progress was made in controlled rolling and controlled cooling technology, which achieved precise control over the heat treatment process of micro-alloyed steel, promoted the application and development of micro-alloyed steel, and fully utilized the strengthening effect of micro-alloyed steel [15]. The literature shows that in the 1990s, significant progress was made in the production technology of micro-alloyed steel. The significant improvement in steel properties has been achieved through the thermo-mechanical control process (TMCP) and the fine addition of micro-alloying elements such as niobium (Nb), titanium (Ti), and vanadium (V). These elements are added to steel at extremely low levels, effectively improving the strength and toughness of the steel through precipitation strengthening and grain refinement mechanisms [16,17,18]. In addition, during the 1990s, micro-alloyed steel was widely used in applications such as construction, heavy-duty structures, and high-pressure pipelines.

The “Micro-alloying 2015” conference [19,20,21,22] explored the significant improvement effects of micro-alloying elements such as vanadium, titanium, niobium, etc. on the strength, toughness, and wear resistance of steel and discussed the potential applications of new alloying elements. The optimization of thermal-mechanical treatment technology (TMT) is also a focus of discussion. By precisely controlling the heating, rolling, and cooling processes, the microstructure and mechanical properties of steel can be effectively improved. The meeting also analyzed the practical applications of micro-alloyed steel in the automotive, construction, and energy fields, emphasized the challenges faced in the context of environmental protection and sustainable development, and proposed directions for future technological development, such as intelligent manufacturing and data-driven material design. Recent studies have shown that the development of micro-alloyed steel is still ongoing, particularly in improving the strength and toughness of steel, optimizing production processes, and developing new application areas [23,24,25]. For example, improving the mechanical properties of steel by controlling TiN precipitation behavior and studying the performance of micro-alloyed steel under extreme conditions. In addition, with the increasing emphasis on environmental friendliness and sustainable development, micro-alloyed steel is regarded as an “environmental protection material” with broad development prospects due to its high efficiency and energy-saving characteristics in the production process [26,27,28].

### 2.2. Application Status of Micro-Alloyed Steel

#### 2.2.1. Application of Titanium Micro-Alloyed Steel

**Figure 1:**
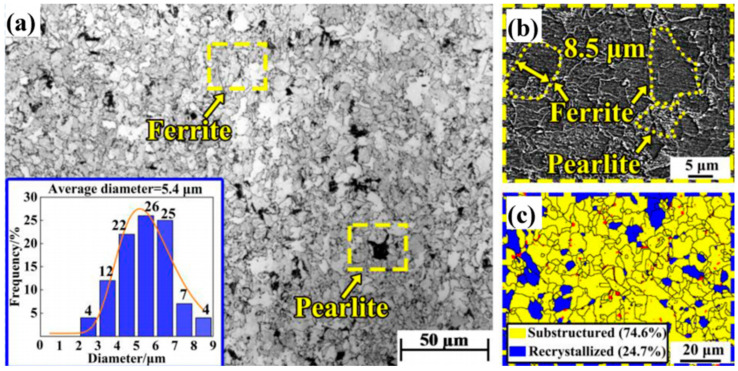
OM (**a**), FESEM (**b**), and EBSD map (**c**) of traditional weathering steel [30].

Titanium is a widely used micro-alloying element at low cost. Many steel companies and research institutes have conducted extensive experimental research and simulations on the microstructure and production process of titanium micro-alloyed steel, and the research results have been applied to practical engineering. The composite of Ti and V elements is currently a hot research topic. Some studies have shown that at high deformation temperatures, as the strain rate increases, the average grain size of austenite first decreases and then increases [29]. Nowadays, researchers have studied the microstructure and performance changes of 700 MPa ultra-low carbon weathering steel by changing the rolling process [30] and obtaining the strengthening mode rules. The microstructure type of steel presents as ferrite + pearlite (as shown in Figure 1). In the microstructure analysis of traditional weathering steel, the ferrite phase is mainly in polygonal form, which constitutes the main microstructure of the steel. These ferrite grains undergo recrystallization and recovery during hot rolling and subsequent coiling processes, forming relatively coarse grain sizes with grain diameters mostly distributed in the range of 4–7 μm, with an average grain size of 5.4 μm. In the ferrite matrix, pearlite exists as a secondary phase, mainly distributed along the grain boundaries of ferrite, with a volume fraction of about 4%. Pearlite is a composite structure formed by alternating layers of ferrite and cementite, typically found in steels with high carbon content. However, in the weathering steel studied in this study, its proportion is relatively low. In terms of precipitates, the precipitates in traditional weathering steel are mainly titanium carbides (TiC), which exist in spherical form with a size distribution between 5 and 40 nm and an average diameter of 18.2 nm. Through transmission electron microscopy (TEM) and energy dispersive spectroscopy (EDS) analysis, it was confirmed that these particles are TiC. These TiC precipitates play a significant strengthening role in steel because they can effectively hinder the movement of dislocations, thereby enhancing the strength of the material. In traditional weathering steel, the proportion of TiC particles smaller than 10 nm is relatively low, which limits its precipitation strengthening effect. In order to ensure that the size of TiC meets the requirements, the cooling rate of Ti micro-alloying solidification should be increased, and the Ti content should be reduced [31]. Jin Y L’s research [32] shows that reducing carbon content can alleviate the grading of banded structures. Although the reduction of carbon content can improve the low-temperature impact toughness of hot-rolled titanium micro-alloyed high-strength steel, it cannot completely solve the problem of poor low-temperature impact toughness. Compared with dislocation strengthening and solid solution strengthening, fine grain strengthening and precipitation strengthening have better strengthening effects, among which the precipitation strengthening of the second-phase particle TiC is the most effective. The literature has improved the strengthening effect by controlling the N and S mass fractions and appropriate processes to obtain more small-sized and dispersed TiC particles.

Scholars [33] have studied the influence of micro-alloying elements Ti and Nb on the microstructure and properties of low-carbon high-strength steel. The research results indicate that due to the different effects of Nb and Ti, the average grain size in Ti-containing micro-alloyed steel is larger than that in Nb-containing micro-alloyed steel. However, there is an abnormal growth of ferrite in Nb steel, which makes the grain size of Nb-containing steel very uneven. Therefore, Ti-containing steel often exhibits better strength and plasticity. This also indicates that for some low-carbon micro-alloyed steels, adding Ti has a higher cost-effectiveness compared to adding the Nb element.

**Figure 2:**
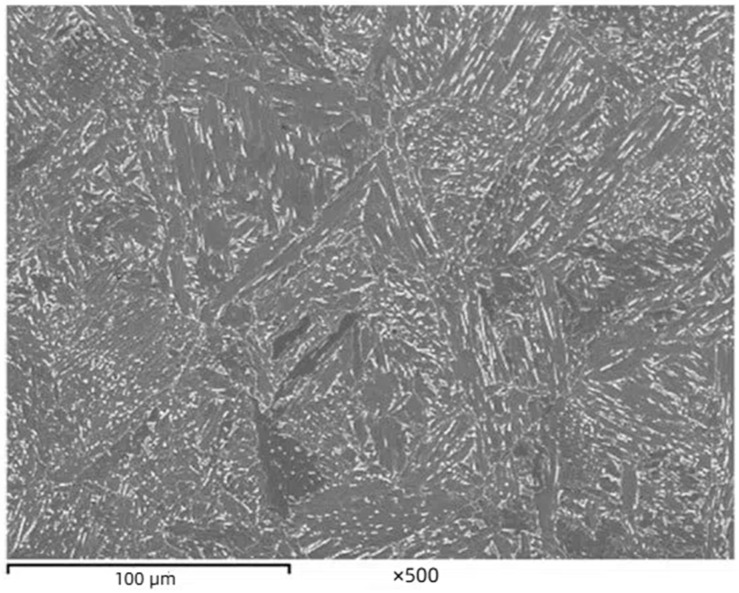
Microstructure of acicular ferrite in steel containing (wt.%): 0.08C-0.21Mo-0.165Ti [37].

Zhang Y [34] developed a 700 MPa high-strength weathering steel using controlled rolling technology and studied its microstructure and mechanical properties. Research has shown that compared to ordinary carbon steel, the relative corrosion rate of titanium steel in Ti-containing steel is less than 55%. High-strength weathering steel with a Ti content of 0.12% has better corrosion performance than titanium steel with a Ti content of 0.155%. The corrosion rust layer of Ti-containing steel is composed of inner and outer rust layers, and the inner rust layer is tightly bonded to the substrate. Excessive Ti content can lead to the formation of large-sized titanium-containing phases, such as TiN, TiS, TiO, etc. The above-mentioned titanium-containing phases consume a large portion of Ti, inhibit the formation of TiC, weaken the precipitation strengthening effect, and have adverse effects on the mechanical properties of steel [35]. Researchers [36] observed that the microstructure of two titanium sheets of steel with different Ti contents consisted of ferrite and a small amount of granular bainite (as shown in Figure 2), and the average grain size of the two Ti-content micro-alloyed sheets of steel was not significantly different. A certain amount of Ti content in high-strength weathering steel can cause it to form nanoscale precipitates with the C element in the steel. The Ti element can also synergistically interact with elements such as Cr, Ni, and Cu in weathering steel [37], forming relatively dense flocculent products that are conducive to preventing corrosive liquids from entering the iron-based structure and achieving strong anti-corrosion effects.

#### 2.2.2. Application Status of Niobium Micro-Alloyed Steel

Compared to titanium, niobium has a more significant inhibitory effect on the recrystallization of austenite, but it is more expensive. The recrystallization of austenite is suppressed, and the nucleation rate of austenite is increased, which is beneficial for refining austenite grains. The role of Nb in micro-alloyed steel depends on the state of Nb and its interactions with other elements or compounds. Nb mainly exists in micro-alloyed steel in the form of Nb solute or the Nb-containing phase. Improving the solid solubility of Nb in steel, controlling the state of Nb in austenite, and promoting the precipitation of the Nb-containing phase can all improve steel properties [38]. The steel used for high-speed railway wheels is specifically engineered to meet stringent requirements for strength, wear resistance, and corrosion resistance. This steel typically exhibits a minimum yield strength of 1000 MPa and a hardness of 400 HV, ensuring exceptional durability under the high stresses experienced during high-speed operations. Studies have shown that through micro-alloying with elements such as vanadium and chromium, the wear resistance is significantly improved, with a reduction in wear rate by up to 30% compared to conventional steels. Additionally, corrosion resistance is enhanced by the formation of a passive film that protects the steel from environmental degradation, as demonstrated by a 50% increase in salt spray test duration without signs of corrosion. These properties are crucial for maintaining the safety and reliability of high-speed rail systems over extended periods of service. Regarding the medium-carbon high-speed railway wheel steel with a carbon mass fraction of 0.47%, Miao C [39] studied the effect of niobium micro-alloying on the austenite reverse-phase transformation of the precursor ferrite pearlite structure. Niobium, as a micro-alloying element in high-speed rail wheel steel, mainly exists in the form of precipitates. During forging and rolling processes, precipitation of niobium already exists, which helps to increase the nucleation rate of austenite and refine austenite grains. As the heating temperature of austenite continues to increase, the finely dispersed precipitates in niobium micro-alloyed wheel steel play an important role in effectively pinning grain boundaries and suppressing austenite coarsening. These precipitates can delay the temperature of austenite mixing in high-speed rail wheel steel, thereby expanding the heat treatment window and improving the applicability of production processes [40]. By adopting appropriate rolling processes, it is possible to obtain austenite structures that undergo deformation before phase transformation and refined room temperature structures with high mechanical strength [41,42,43]. In the process of increasing the heating temperature of austenite, the small-dispersed precipitates present in the wheel steel have a significant effect on pinning grain boundaries and inhibiting austenite grain coarsening, which can increase the austenite mixed crystal temperature of high-speed wheel steel from 880 °C to 960 °C. The synergistic effect of niobium and molybdenum on hardenability in high-strength medium-carbon steel was subjected to quenching and subsequent tempering treatment. By optimizing mechanisms such as solution hardening, unit size refinement, strain hardening, fine-precipitation hardening, and the effect of solution carbon, the mechanical properties of the alloy have been improved [44]. In general, using the tempering process to treat martensitic structures can improve the toughness and ductility of steel [45,46]. Zhang X et al. [47] studied low-alloy high-strength steels with different Nb contents and analyzed the effect of Nb content on the strengthening mechanism of low-carbon steel. Research has shown that the strengthening methods for low-carbon steel vary depending on the niobium content. Low-niobium steel mainly relies on fine-grain strengthening to enhance its properties, while high-niobium steel mainly relies on fine-grain strengthening and precipitation strengthening. With the increase of niobium content, the precipitation of niobium carbonitride increases, and the precipitation strengthening effect is significantly enhanced, increasing the strength of the steel. Steel with a niobium content of 0.036% can refine the grain size to about 5 µ m, thereby achieving the desired fine grain strengthening effect in general situations.

In the context of Nb micro-alloyed steel, the refinement of niobium (Nb) solute and its correlation with grain size are crucial in the heat treatment process. The term “refinement” in this context refers to the reduction of solute particle size to the nanoscale, which significantly impacts the steel’s microstructure and properties. “Grain size” denotes the dimensions of individual crystals within the steel matrix, with a finer grain size generally associated with improved strength and toughness. For instance, the addition of Nb in micro-alloyed steels leads to the formation of fine Nb (C, N) precipitates that effectively pin the grain boundaries and inhibit grain growth during heat treatment. This results in a refined microstructure, with grain sizes typically in the range of 5–10 μm, significantly smaller than those found in conventional steels. The refinement of Nb solute and the resulting reduction in grain size have substantial effects on the mechanical properties of the steel, as evidenced by the enhanced strength and ductility observed in micro-alloyed steels compared to their conventional counterparts [48]. We reasonably determine the austenitization temperature and time based on the Nb content during the austenitization process to ensure the normal growth of austenite grains. The controlled rolling process can retain the strain energy in austenite, thereby increasing the nucleation rate and refining the grain size. By adjusting the heat treatment and isothermal annealing process parameters, the supersaturation precipitation of Nb (C, N) in ferrite or bainite can be promoted, and the precipitation strengthening generated by the precipitation of nanoscale Nb (C, N) can improve the strength of micro-alloyed steel [49,50,51].

#### 2.2.3. Application Status of Titanium–Niobium Micro-Alloyed Steel

Zhu WT, Liu W [52,53], and others studied the effect of final rolling temperature on the microstructure and properties of TMCP low-alloy titanium–niobium bainitic steel. They found that as the final rolling temperature decreased, the strength and toughness of the steel first increased and then decreased. When the finishing rolling temperature is 870 °C, fine bainite flat noodles and a small amount of martensite structure are obtained, and Ti and Nb precipitates are dispersed on the bainite flat noodles, obtaining the best performance. As the temperature decreases, the deformation bands, dislocations, and substructures generated by rolling provide more nucleation sites for precipitates, making them more dispersed and precipitated. Rajput SK [54] used the Gleebe3500 thermal simulation test machine to study the continuous cooling transformation law of Nb Ti micro-alloyed test steel under deformed and undeformed conditions. The steel can obtain a bainite structure in a large cooling rate range of ~2–50 °C/s. With the increase of the cooling rate, the amount of granular bainite in the structure decreases, and the amount of flat noodles bainite increases. Simultaneously, deformation promotes phase transformation, which is beneficial for the formation of new phases in austenite. The final rolling temperature has a significant impact on the mechanical properties of Ti-Nb micro-alloyed steel. As the final rolling temperature continues to decrease, the ferrite grains are significantly refined, resulting in a significant increase in the effect of fine-grain strengthening [55,56].

Titanium–niobium micro-alloyed steel is also widely used in the shipbuilding industry. After familiarizing themselves with the austenite recrystallization law of marine steel plates, researchers [57] developed a 60 mm-thick marine special steel plate with high strength, good welding performance, good resistance to layer tearing, and good impact toughness by controlling the rolling process, cooling process, and normalizing heat treatment process, which meets the requirements of thick specifications, high strength, and high toughness. The strain aging of steel is influenced by many factors such as the composition of the steel plate, smelting method, residual stress during rolling, plastic processing, and welding residual stress [58,59,60]. Controlling the content of residual elements S, N, O, and Si in steel can reduce the aging impact energy of steel, ensure sufficient deoxidation during smelting, reduce the total oxygen of steel, and minimize oxide impurities. Adding an appropriate amount of Ti, usually around 0.015%, can improve the aging impact toughness of steel [61]. Roll the steel in the recrystallization zone and nonrecrystallization zone. During the heating process of austenite, by controlling the heating temperature and heating time, the Nb and Ti carbonitrides in the steel are fully dissolved in the austenite while also preventing abnormal growth of the original austenite grains [62,63].

**Figure 3:**
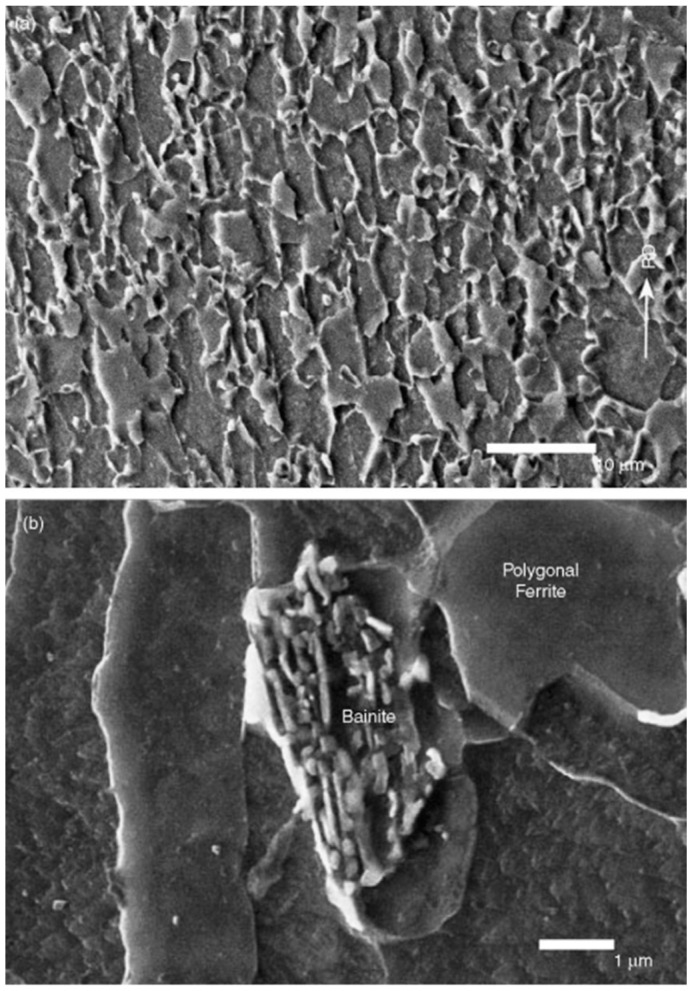
(**a**) Low-magnification light micrograph and (**b**) relatively high-magnification scanning electron micrograph of 770 MPa Nb–Ti micro-alloyed hot rolled steels [64].

The current application status of Ti-Nb micro-alloyed steel is reflected in its excellent combination of strength and toughness, successfully achieving a yield strength of up to 770 MPa while maintaining good plasticity and toughness. As shown in Figure 3 [64], the microstructure of this steel is characterized by fine ferrite grains and approximately 10% bainite, which are achieved through controlled rolling and cooling processes. This material has been commercially applied in fields such as automotive, construction, and mechanical engineering, as it can reduce material usage without sacrificing performance while lowering production costs and improving energy efficiency. However, research on Ti-Nb micro-alloyed steel is ongoing, aiming to further enhance its toughness and economy to meet a wider range of industrial demands and drive continuous progress in material properties.

### 2.3. Application Issues of Titanium–Niobium Micro-Alloyed High-Strength Steel

When the Nb content reaches a certain level, adding Nb again has little effect on the strength of the micro-alloyed steel. At this point, adding Ti can obtain higher-performance micro-alloyed steel. When the Ti content increases, a large number of small precipitates will be produced, which have a strong precipitation strengthening effect and significantly improve the yield strength. The increase in Nb and Ti content is equivalent to the strengthening effect of increasing Ti content alone, and the increase in strength is mainly due to the increase in Ti content, rather than the increase in Nb content [64,65,66]. Compared to niobium, titanium is considered to have higher chemical activity, which affects its reactivity and precipitation behavior in steel matrices. In addition, titanium is more cost-effective. According to a report from the US business analysis website Businessanalytiq, the average price of titanium in 2024 is about USD 4.6 per kilogram, much lower than the USD 41.8 per kilogram price of niobium, according to the latest market report. These price points will greatly affect the economic feasibility of using these micro-alloying elements in steel production. In the research of titanium–niobium micro-alloyed steel, it is more inclined to obtain micro-alloyed steel with target properties by changing the Ti content. Due to the differences in micro-alloying elements and processing techniques of micro-alloyed steel, the issue of Ti and Nb element content has not been well resolved. The processing of titanium–niobium micro-alloyed steel also has inherent metallurgical disadvantages of other steel grades, such as residual austenite, residual stress, quenching cracks, and deformation [67,68].

When the mixed crystal phenomenon occurs, the low-temperature impact energy of the steel plate will be poor, and the stability of low-temperature toughness will also decrease accordingly. In the actual production process, the rolling deformation during the rough rolling stage should be greater than 66.7% to avoid the occurrence of mixed crystal phenomenon [69]. During the precision rolling stage, a smaller amount of deformation should be used to facilitate grain refinement during the cooling phase-transition process, while a smaller reduction is beneficial for controlling the shape of the strip steel. With the increase of high-temperature reduction, austenite recrystallization becomes more complete, and the grain size of the finished product structure becomes finer. Therefore, under the premise of equipment rolling capacity, the high-temperature deformation of rough rolling passes should be increased as much as possible [70,71,72].

Titanium–niobium micro-alloyed steel faces multiple challenges in terms of secondary processing performance, mainly reflected in the optimization of steel properties during welding, forming, and heat treatment processes. Titanium–niobium micro-alloyed steel is prone to performance degradation during welding, and high temperatures can cause the dissolution or redistribution of carbonitride precipitates of titanium and niobium, affecting the strength and hardness of the steel. In addition, the performance of the welding heat-affected zone (HAZ) significantly decreases due to grain growth and precipitation changes [73]. In high-strength duplex steels such as DP980, the addition of niobium improves the strength of the material, but the dissolution of precipitates during welding can weaken the heat-affected zone [74]. During the stamping process of titanium–niobium micro-alloyed steel, due to its high strength, the ductility and equilibrium mechanical properties of the material are key issues. For example, during the bending process of high-strength duplex steel, significant rebounds are prone to occur, which affects the dimensional accuracy and shape of the parts. In addition, stress concentration areas are prone to cracking during the forming process, which needs to be improved by optimizing the forming speed and lubrication conditions. The recrystallization and phase transformation behavior of titanium–niobium micro-alloyed steel during heat treatment is complex [75]. During recrystallization annealing, the precipitation of titanium and niobium can affect grain growth, and controlling the heat treatment process reasonably is the key to improving material properties [76]. For example, during the heat treatment process, by precisely controlling the temperature and time, the size and distribution of precipitates can be optimized, improving the overall performance of the material. However, high-temperature treatment may also lead to the re-dissolution or re-precipitation of precipitates, affecting the final performance [77].

## 3. Factors Affecting Organizational Performance

### 3.1. Strengthening Methods

There are five strengthening methods for titanium–niobium micro-alloys, with fine grain strengthening and precipitation strengthening playing relatively key roles, followed by solid solution strengthening and dislocation strengthening.

The yield strength of micro-alloyed steel can be calculated by Equation (1) [78]:

(1) $$\sigma_{y}=\sigma_{o}+\sigma_{g}+\sigma_{p}+\sigma_{s}+\sigma_{\rho }$$

In Equation (1), $\sigma_{y}$ is the yield strength, measured in MPa; $\sigma_{o}$ is the lattice friction stress, approximately 50 MPa; $\sigma_{g}$, $\sigma_{p}$, $\sigma_{s}$, and $\sigma_{\rho }$ are the increments of fine-grain strengthening, precipitation strengthening, solid solution strengthening, and dislocation strengthening, respectively.

The increment of fine-grain strengthening can be calculated by Equation (2) [79]:

(2) $$\sigma_{g}=k_{\mathrm{hp}}{\bar{d}}^{-1/2}$$

In Equation (2), k_hp_ is the interaction constant of the element in the range of ~15.1–18.1 N/mm^3/2^, and $\bar{d}$ is the effective grain size.

The increment of precipitation strengthening can be calculated by Equation (3) [80]:

(3) $$\sigma_{p}=k_{p}f_{p}(\Delta G{)}^{2}$$

Equation (3), represents the volume fraction of $f_{p}$ precipitate in %, $k_{p}$ is the precipitation strengthening coefficient, and ΔG is the difference in shear modulus between the precipitate phase and the matrix.

The increment of solid solution strengthening can be calculated by Equation (4) [81]:

(4) $$\sigma_{s}=\Sigma_{i}k_{i}x_{i}$$

In Equation (4), x_i_ represents the mass fraction of the i-th element, expressed in %; k_i_ is the solid solution strengthening coefficient of the i-th element.

The increment of dislocation strengthening can be calculated by Equation (5) [82]:

(5) $$\sigma_{\rho }=k_{\rho }\sqrt{\rho }$$

In Equation (5), $k_{\rho }$ represents the dislocation strengthening coefficient, and ρ represents the dislocation density.

(1) Precipitation strengthening

**Figure 4:**
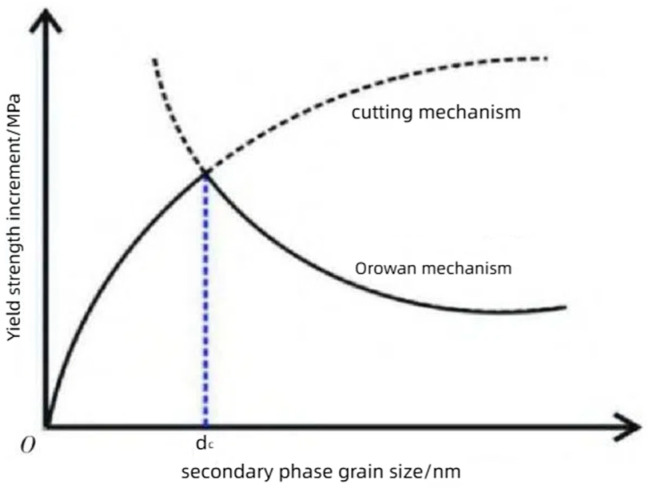
The influence of second-phase grain size on strengthening mechanism.

Under the same process, the yield strength of titanium–niobium steel is always higher than that of low-carbon steel, mainly due to the significant precipitation strengthening effect of (Nb, Ti) (C, N) precipitated during the isothermal process of micro-alloyed steel [83]. The precipitation strengthening of titanium–niobium micro-alloys is largely influenced by the volume fraction of its second-phase inclusions and the size of the precipitated particles, and the key to precipitation strengthening lies in the small, dispersed second phase hindering the migration of dislocations [84]. The influence of the second-phase grain size on the precipitation strengthening mechanism is shown in Figure 4. When the size of the second-phase grains does not reach the critical size (dc), it is mainly due to the cutting mechanism [85]. When the size of the second-phase grains is larger than the critical size (dc), it is mainly due to the bypassing mechanism (i.e. Orowan mechanism) [86].

When nanoscale second-phase particles precipitate in steel, the particle size is small, and the dislocation motion will directly cut through the second phase, achieving the effect of precipitation strengthening. When the size of the second-phase particles is large, dislocation motion will bypass the second-phase particles to form dislocation loops, and the precipitation strengthening effect will decrease with the increase of the second-phase particle size [87,88,89]. The precipitation hardening of nanoscale Ti and Nb carbide particles is the main factor that increases the yield strength of high-strength micro-alloyed steel.

The effect of precipitation strengthening is related to the size and quantity of precipitation particles, and the effect of precipitation strengthening is calculated by Formula (6):

(6) $$\Delta \sigma =\frac{5.9f^{1/2}}{\bar{x}}\mathrm{lg}\left(\frac{\bar{x}}{2.5\times 10^{-4}}\right)$$

In Equation (6), Δσ is the increase in strength and f is the volume fraction of carbonitride.

$\bar{x}$ is the truncated diameter of precipitated phase particles on the slip plane m, calculated from $D(2/3{)}^{1/2}$, and D is the average diameter of the particles. From this, it can be seen that the smaller the average particle size, the more precipitation occurs, and the more obvious the precipitation strengthening effect.

Based on the existing precipitation strengthening theory, we believe that the precipitation kinetics of micro-alloying elements under nonisothermal conditions must be reevaluated, as this may have a decisive impact on the material’s final properties.

(2) Fine-grained strengthening

Fine-grain strengthening is one of the main strengthening methods for micro-alloyed steel, and its strengthening mechanism is that as the material grains become smaller, the grain boundary area increases, accompanied by disordered and differently oriented grains on both sides of the grain boundary, and the arrangement of atoms at the grain boundary is very disordered [90,91]. For the fine-grain strengthening of micro-alloyed steel, the micro-alloying elements Nb and Ti are usually dissolved in high-temperature austenite, and then the carbon–nitrogen compounds of the micro-alloying elements are precipitated according to the appropriate size through controlled rolling and cooling processes [92].

The carbonitrides of titanium and niobium, two micro-alloying elements, generally precipitate at grain boundaries, which, to some extent, hinder the movement of grain boundaries. And at high temperatures, it can also suppress the recrystallization of austenite grains, avoiding the coarsening of austenite grains. The micro-alloying elements are dissolved in austenite precipitate as finely dispersed second-phase particles in ferrite or pearlite crystals, or at grain boundaries during subsequent controlled rolling and controlled cooling processes. Titanium and niobium, two micro-alloying elements, have a certain inhibitory effect on the recrystallization of original austenite grains, and niobium has a stronger inhibitory effect on the recrystallization of original austenite grains than titanium [93,94,95]. The second-phase grains generated by the niobium element play a pinning role in grain boundaries, which can effectively suppress the recrystallization of austenite grains. When combined with titanium elements, the effect of refining grains is very significant.

According to the knowledge of metallurgy, the relationship between the yield strength and grain size of materials follows the Hall–Petch formula [96].

$$\sigma =\sigma_{0}+{\mathrm{kd}}^{-\frac{1}{2}}$$

The d is the diameter of ferrite grains, and $\sigma_{0}$ is the frictional force acting on dislocations. d not only represents the grain diameter but also represents the size of subgrains and polygonalized lines in metals with subgrain structures. In pearlite, d can represent the interlayer spacing.

According to the Hall–Petch formula, the yield strength is inversely proportional to the 1/2 power of the grain size. Therefore, under the same conditions, the smaller the grain size, the greater the yield strength of the material. In addition, due to the small grain size, the number of grains in the same volume of crystal increases, the area of grain boundaries in the same volume increases, and the resistance of dislocations that continuously slip the same distance increases. When the material is subjected to stress deformation, the dispersion effect of grains on the deformation amount makes the deformation degree of each grain small and uniform, which can significantly reduce the locally concentrated stress value and hinder the generation and development of microcracks [97,98]. Simultaneously refining the grain size and introducing a large number of grain boundaries can effectively hinder the generation and development of cracks, further improving the toughness and plasticity of the crystal. Therefore, by combining the characteristics of Nb and Ti micro-alloying elements, adding appropriate Nb and Ti elements, and controlling the subsequent heat treatment process, the strength and ductility of steel can be simultaneously improved.

In practical applications, micro-alloyed steel typically combines these two strengthening mechanisms to achieve superior mechanical properties. By optimizing the types and contents of micro-alloying elements, a good balance between precipitation strengthening and grain refinement strengthening can be achieved in steel.

### 3.2. Effects of Components

Compared to adding a micro-alloying element with Ti as the main component and Nb as the auxiliary component, Ti-Nb micro-alloyed steel can achieve both fine grain strengthening and precipitation strengthening, making it easier to achieve a good match between high strengthening and excellent ductility of micro-alloyed steel. A high-strength weathering steel with a yield strength of 550 MPa was developed through the composition design of Nb and Ti content, combined with controlled rolling and cooling processes [99]. It has good strength plasticity matching, elongation greater than 22%, and a ductile-brittle transition temperature below −80 °C. The microstructure of high-strength weathering steel consists of ferrite, pearlite, and granular bainite, with high-density dislocations and nanoscale niobium titanium composite precipitates within the grains.

There are two reasons for the increase in steel strength in the content of micro-alloying elements. The first is the fine-grain strengthening effect obtained by the carbon–nitrogen precipitates of micro-alloying elements preventing grain growth [100]. The second is the precipitation strengthening effect caused by the nanoscale precipitation of (Ti, Nb) (C, N) particles in the matrix, which hinders the movement of dislocations [101].

Titanium and niobium are both strong carbonitride-forming elements. Ti will preferentially combine with N to form TiN. Due to the relatively large particle size of TiN and the fact that it does not dissolve under most high-temperature conditions, TiN can increase the strength of the welding zone [102]. In addition, TiN precipitation at high temperatures helps to prevent the growth of austenite grains. When the Ti content is high, in addition to forming TiN, the remaining Ti will combine with C in the steel to form smaller TiC particles, which can play a role in precipitation strengthening. Generally speaking, the Ti content is maintained within the range of ~0.06–0.12 wt% to ensure effective grain refinement and precipitation strengthening effects. When the Ti content is below this range, it may not be sufficient to form sufficient TiN and TiC particles, resulting in insufficient grain refinement and an insufficient precipitation strengthening effect, which will reduce the strength and toughness of the material. In addition, insufficient titanium content may weaken welding performance and affect the strength of welded joints. On the contrary, excessive titanium content may lead to the excessive formation of TiN inclusions, which may become the source of stress concentration, thereby reducing the toughness of the material, especially under low-temperature conditions. Meanwhile, excessive TiC particle aggregation may weaken the precipitation strengthening effect and increase material costs, while the marginal benefits of performance improvement may not be significant. Therefore, controlling the Ti content within an appropriate range can balance the strength, toughness, and cost-effectiveness of the material while avoiding processing difficulties and performance degradation issues, ensuring that titanium–niobium micro-alloyed high-strength steel exhibits excellent comprehensive performance in various industrial applications [103,104]. Trace Nb in steel can inhibit the recrystallization of deformed austenite, prevent the growth of austenite grains, and improve the strength and toughness of steel. In addition, the precipitation of Nb (C, N) during the cooling process can play a role in precipitation strengthening, improving the mechanical properties of steel. Considering the solid solubility product of Nb and related elements in austenite and ferrite, as well as the content of Ti, N, S, C, and other elements in steel, and combining with the ideal chemical ratio of related compounds, the Nb content control range is ~0.03–0.08 wt% [105,106,107].

### 3.3. Effects of Thermo-Mechanical Controlled Processing

**Figure 5:**
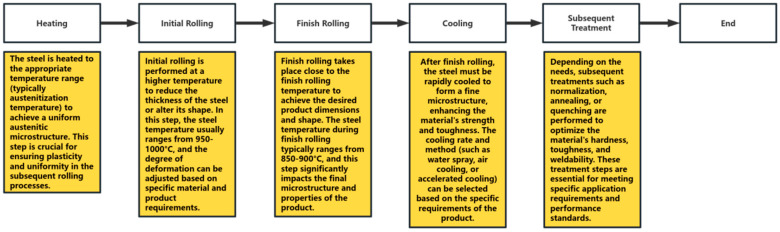
Example diagram of the traditional TMCP process.

TMCP, also known as thermo-mechanical controlled processing, originated in the 1980s and is a significant technological advancement in steel rolling technology. In the production of micro-alloyed high-strength steel, the traditional TMCP process principle [108] generally involves refining austenite grains and controlling the cooling rate of the post-rolling cooling process to achieve phase-transformation strengthening through “low-temperature large reduction” and “micro-alloying” rolling in the austenite recrystallization zone and nonrecrystallization zone (for example, Figure 5). The core idea of controlling rolling is actually to control the hardening state of austenite. During the rolling process, the hardened austenite prepares for the grain refinement required for subsequent phase transformation [109]. Sometimes it is also possible to meet the process requirements by adding alloying elements, such as adding the Nb element. After adding the Nb element, the recrystallization temperature of austenite increases, which can keep austenite in the amorphous zone and increase its deformation, thereby achieving austenite hardening [110,111]. The core idea of controlling cooling is to control the austenite transformation process in the hardened state, refine the ferrite grains, and further improve the various properties of the material. The main problem with controlled cooling technology now is the large residual stress caused by uneven cooling of materials after high-speed cooling. For example, the current quenching process, as the limit of controlled cooling, has a significant effect on improving the hardness of steel. However, the problem of uniform cooling caused by quenching has been troubling people.

Although traditional controlled rolling and controlled cooling technology is relatively mature and has significant advantages in production efficiency, process cost, and product quality compared to conventional hot rolling processes, its limitations are also very prominent [112]. Low-temperature high pressure “refers to the rolling deformation treatment of steel using a larger pass reduction near the phase transition temperature”. This method can increase the interfacial area-per-unit volume, thereby improving the nucleation rate of phase transition [113]. Under “low temperature and high pressure”, the load on the rolling mill increases sharply, making it prone to accidents such as rolling jamming, which inevitably leads to plate shape problems. So, a new generation of TMCP technology was developed, breaking through the limitations of traditional TMCP technology in treating micro-alloyed steel [114]. The core concept of the new generation TMCP technology is to continuously roll and deform micro-alloyed steel in a higher temperature range to obtain austenite with high-strain energy and then perform reasonable ultra-fast cooling according to the application needs of micro-alloyed steel, finally obtaining high-performance steel [115,116].

Due to the significant inhibition of austenite recrystallization by Nb and the inhibition of austenite grain growth by Ti, rolling followed by air cooling and, finally, ultra-fast cooling can be used in the process of processing micro-alloyed high-strength steel [117]. The obtained steel will mainly consist of fine-grained polygonal ferrite, with a small amount of pearlite or bainite, and a large amount of Ti and Nb carbides will precipitate, resulting in a significant strengthening effect. The precise control of rolling parameters plays a decisive role in the microstructure and mechanical properties of Ti-Nb micro-alloyed low-alloy steel during the rolling process. The decrease in final rolling temperature tends to suppress the dynamic recrystallization of austenite, promote the formation of fine grains, and enhance the precipitation strengthening effect of Ti and Nb carbonitrides [118,119]. The increase in cooling rate helps to fix the microstructure introduced by rolling and promotes the uniform precipitation of small Ti and Nb carbonitrides, thereby enhancing the strength and toughness of the material. The increase in rolling deformation increases the dislocation density of austenite, promotes grain refinement, and further enhances the strength of the material. However, excessively high rolling speeds may lead to dynamic recrystallization, affecting grain size and distribution and thereby adversely affecting material properties [120]. The rolling passes, and the interval time between passes, have a significant impact on the precipitation kinetics of Ti and Nb carbonitrides and the recrystallization behavior of austenite and require precise control to optimize the microstructure of the material. In addition, the heat treatment process after rolling, such as normalizing or annealing, is equally crucial for the microstructure and properties of Ti and Nb micro-alloyed steels. Appropriate heat treatment can optimize the size and distribution of Ti and Nb carbonitrides, achieving the optimization of material properties [121,122]. Therefore, by comprehensively considering and precisely controlling the rolling parameters, the microstructure of Ti-Nb micro-alloyed low-alloy steel can be effectively controlled, achieving fine grain strengthening and precipitation strengthening, thereby obtaining excellent comprehensive mechanical properties.

## 4. Development Prospects of Titanium–Niobium Alloys

In this review, we delve into the research progress of titanium–niobium micro-alloyed high-strength steel, focusing on analyzing the effects of metallurgical processes and second-phase particles on material properties. We found that through the mechanisms of fine-grain strengthening and precipitation strengthening, titanium–niobium micro-alloying elements significantly improved the strength and toughness of steel. Although some progress has been made, some problems and challenges remain, such as the degradation of welding performance, stability of precipitates at high temperatures, and crack sensitivity during the forming process. In response to these issues, future research should focus on the following key areas: further investigation of the mechanisms of precipitation strengthening and grain-refinement strengthening, particularly the influence of second-phase particles on dislocation motion; optimize rolling and cooling processes to achieve fine control of austenite grains and uniform distribution of second-phase particles; by using alloy design and processing techniques, the characteristics of TiN, TiC, NbC, and other particles can be precisely controlled to achieve the best strengthening effect; develop new welding technologies, improve heat treatment processes, and optimize forming parameters to solve practical application problems; evaluate the energy efficiency of titanium–niobium micro-alloyed high-strength steel in the production process; and explore methods for material recycling and scrap disposal. In summary, research on titanium–niobium micro-alloyed high-strength steel will continue to make progress in deepening mechanism understanding, process optimization, particle control, application problem solving, and environmental sustainability to achieve wider applications and better performance in future industrial applications.

This article takes Ti-Nb micro-alloyed high-strength steel as the main research object, elaborates on the development of Ti-Nb micro-alloyed high-strength steel, and analyzes the influence of micro-alloyed composition and controlled pinning and cooling processes on the properties of micro-alloyed steel based on the strengthening mechanism. The research and development of future titanium–niobium micro-alloyed steel can focus on the following four points:

(1) In-depth research on strengthening mechanisms

Future research should continue to deepen the understanding of precipitation strengthening and grain refinement mechanisms in titanium–niobium micro-alloyed high-strength steel. Especially for the size, distribution, and mechanism of the influence of the second-phase particles on dislocation motion, more in-depth experimental and theoretical analysis is needed. In addition, the roles of solid solution strengthening and phase-transformation strengthening cannot be ignored, and further exploration is needed on how these strengthening mechanisms work together to achieve optimal material properties.

(2) Optimization of metallurgical processes

The performance of titanium–niobium micro-alloyed high-strength steel depends not only on the alloy composition but is also significantly influenced by metallurgical processes. Future research needs to focus on how to optimize the microstructure of materials by controlling process parameters such as rolling and cooling. For example, by adjusting the final rolling temperature, cooling rate, and heat treatment system, the size and distribution of austenite grains can be effectively controlled. In addition, promoting the precipitation of finely dispersed second-phase particles can improve the strength and toughness of the material.

(3) Control of second-phase particles

The second-phase particles play a crucial role in titanium–niobium micro-alloyed high-strength steel. Future research should focus on controlling the formation, size, morphology, and distribution of these particles to achieve optimal precipitation strengthening effects. This may involve precise control of the precipitation behavior of particles such as TiN, TiC, and NbC, and exploring how to adjust the characteristics of these particles through alloy design and processing techniques.

(4) Solution to application problems

Some problems faced by titanium–niobium micro-alloyed high-strength steel in practical applications, such as the degradation of welding performance, dissolution or redistribution of precipitates at high temperatures, and crack formation during the forming process, need to be further studied to solve. This may include developing new welding techniques, improving heat treatment processes, and optimizing forming process parameters.

(5) Environmental benefits and sustainability

With the increasing emphasis on environmental protection and sustainable development, future research should also consider the environmental benefits of titanium–niobium micro-alloyed high-strength steel, including its energy efficiency in the production process, the possibility of material recycling, and the disposal methods after the end of its service life.

In summary, the future development of titanium–niobium micro-alloyed high-strength steel will focus on a deep understanding of strengthening mechanisms, precise control of metallurgical processes, precise control of second-phase particles, effective solutions to application problems, and comprehensive consideration of environmental benefits and sustainability. Through an in-depth exploration of these research directions, titanium–niobium micro-alloyed high-strength steel is expected to play a greater role in future industrial applications.
